# Cardioplegia between Evolution and Revolution: From Depolarized to Polarized Cardiac Arrest in Adult Cardiac Surgery

**DOI:** 10.3390/jcm10194485

**Published:** 2021-09-29

**Authors:** Alessandra Francica, Filippo Tonelli, Cecilia Rossetti, Ilaria Tropea, Giovanni Battista Luciani, Giuseppe Faggian, Geoffrey Phillip Dobson, Francesco Onorati

**Affiliations:** 1 Division of Cardiac Surgery, University of Verona Medical School, 37126 Verona, Italy; filipp0tonelli@gmail.com (F.T.); rossetticeci93@gmail.com (C.R.); ilariatropea1991@gmail.com (I.T.); giovanni.luciani@univr.it (G.B.L.); giuseppe.faggian@univr.it (G.F.); francesco.onorati@univr.it (F.O.); 2 Heart and Trauma Research Laboratory, College of Medicine and Dentistry, James Cook University, Townsville 4811, Australia; geoffrey.dobson@jcu.edu.au

**Keywords:** myocardial protection, polarizing cardioplegia, adenosine-lidocaine-magnesium

## Abstract

Despite current advances in perioperative care, intraoperative myocardial protection during cardiac surgery has not kept the same pace. High potassium cardioplegic solutions were introduced in the 1950s, and in the early 1960s they were soon recognized as harmful. Since that time, surgeons have minimized many of the adverse effects by lowering the temperature of the heart, lowering K^+^ concentration, reducing contact K^+^ time, changing the vehicle from a crystalloid solution to whole-blood, adding many pharmacological protectants and modifying reperfusion conditions. Despite these attempts, high potassium remains a suboptimalway to arrest the heart. We briefly review the historical advances and failures of finding alternatives to high potassium, the drawbacks of a prolonged depolarized membrane, altered Ca^2+^ intracellular circuits and heterogeneity in atrial-ventricular K^+^ repolarization during reanimation. Many of these untoward effects may be alleviated by a polarized membrane, and we will discuss the basic science and clinical experience from a number of institutions trialling different alternatives, and our institution with a non-depolarizing adenosine, lidocaine and magnesium (ALM) cardioplegia. The future of polarized arrest is an exciting one and may play an important role in treating the next generation of patients who are older, and sicker with multiple comorbidities and require more complex operations with prolonged cross-clamping times.

## 1. Introduction

### 1.1. Dawn of Cardiac Surgery: Making the Impossible a Reality

*“No surgeon who wished to preserve the respect of his colleagues would ever attempt to suture a wound of the heart”*

Theodor Billroth (1881) [1]

For many centuries, wounds of the heart were thought to be fatal and untreatable. Whether Billroth made the above statement is highly debatable [1]. However, there was strong belief among leading authorities that there should be no surgical exploration or intervention anywhere near the heart. This all changed in 1891, when Henry Dalton repaired the first pericardial wound in human in St. Louis, MO, USA [2], followed by similar operations performed by Daniel Hale Williams in Chicago (1893), Axel Cappelen in Norway (1895), Ludwig Rehn in Germany (1896) and Antonio Parrozzani in Italy (1898) [3,4]. The tenacity and courage of these early surgical innovators was supported by a growing number of experimental studies, including those on dogs by Italian Simplicio Del Vecchio (1895) [1]. Within 10 years, there were no fewer than 124 recorded examples of operations on cardiac stab wounds, with a remarkable success rate of 40%, which directly challenged the critics and their ethical concerns [5].

### 1.2. Two Revolutions: Hypothermia and the Heart-Lung Machine

Two decades later, in 1923, the first closed-heart surgery was performed by Cutler and Levine in Boston, treating a mitral stenosis with commissurotomy [6]. The transitioning of these procedures to open-heart surgery took another 35 years and many challenges. A major challenge was protecting the brain and heart during circulatory arrest to allow the surgeon to successfully perform the operation. The answer came from a series of step-by-step laboratory and clinical investigations with: (1) the introduction of ice-packs to cool the patient, and (2) the invention of the heart–lung bypass [7]. Hypothermia was the insight of Wilfred Bigelow from his observations of natural hibernators. Bigelow showed that low temperature in dogs could lower metabolism and potentially widen the ‘safe’ window of circulatory arrest from around 8 min to 30 min [8]. As a result of Bigelow’s work, hypothermia was soon widely adopted by surgeons for many interventions. In 1952 John Lewis and his team appear to be among the first to use hypothermia in humans to successfully repair an atrial septal defect [9]. However, cooling by itself was not sufficient for whole-body protection during cardiac surgery, and in the following year, John Gibbon performed the first open-heart procedure using his heart–lung machine (cardiopulmonary bypass, CPB) [10]. This was a revolutionary step and transformed the practice of open-heart surgery from a possibility to a reality. Gibbon never performed open-heart surgery again, preferring to work with other surgeons, such as John Kirklin at the Mayo Clinic, to continue to develop his machine. In 1955, Kirklin performed the first series of open-heart operations using a modified pump-oxygenator that was coupled with Gibbon’s heart–lung machine that profoundly increased its efficiency [11]. Today, CBP is almost exclusively used for every cardiac surgery operation worldwide.

Despite the revolutionary advances of introducing hypothermia and the Mayo–Gibbon heart–lung machine, there were still problems. Almost all early attempts to correct structural heart diseases were performed on a beating heart or cold fibrillating perfused heart, and clearly the myocardium was not optimally protected [12,13,14]. This shortcoming meant surgeons had to rush their operations and the mortality rates were high. Enter the era of high potassium cardioplegia.

### 1.3. Cardioplegia: The Third Revolution

The term cardioplegia (cardio, “heart” and plegia, “paralysis”) was first introduced by Lam in 1957 [15]. The idea was not new, as British clinician and physiologist Sidney Ringer had published a series of experiments in 1883 showing potassium (K^+^) induced “diastolic arrest” in the frog heart [16]. Although Lam and colleagues used an intraventricular injection of KCl to induce cardiac arrest in hypothermic dogs, he quickly abandoned the experiments because of the occurrence of ventricular fibrillation and myocardial ischemic-reperfusion injury [15,17]. The source of adverse events was believed to be chloride, so Melrose tested the citrate salt of K^+^, and he successfully performed the first cardioplegic arrest in a canine model of CPB. The heart arrested suddenly and remained electrically quiescent for about half an hour, and then resumed its activity and functionality without arrhythmias or myocardial injury [18]. Melrose proposed that high K^+^ citrate could make human cardiac surgery safer by inducing “elective reversible cardiac arrest” [19]. The “Melrose Technique”, as it became known, made a huge contribution to cardiac surgery as a strategy for creating a bloodless field that could allow for surgical accuracy and prevent myocardial injury.

Despite Melrose enthusiasm, several experimental and human studies soon showed that hyperkalemic solution could lead to left ventricular dysfunction, refractory ventricular fibrillation, and cardiac cell death, which was linked to potassium-related cytotoxicity [20,21,22,23,24,25,26]. With so much controversy, hyperkaliaemic cardioplegia was abandoned for many years [22]. Accordingly, alternative methods were sought, including topical and profound hypothermia [27,28,29,30]. Unfortunately, these methods had no better results and resulted in so-called “stone heart” [31]. Another alternative was devised by Bretschneider in 1964, who suggested a Histidine–Tryptophan–Ketoglutarate (HTK) solution. While originally designed for cardiac surgery, it was not widely adopted and began to be used for other purposes, such as preserving liver, kidney and pancreas for transplantation [32,33,34,35].

Some years later, in the mid-1970s, cardioplegic solutions with a high-to-moderate potassium concentration were reintroduced, both in the USA and UK. Among them, St. Thomas’ Hospital (STH) cardioplegia was introduced by Hearse and Braimbridge in London [36,37,38]. They developed two solutions based on different K^+^ concentrations (20 mmol/L in STH solution no 1 and and 16 mmol/L in STH solution no 2). Both contained elevated magnesium (16 mmol/L), and normal Ca^2+^ concentrations, with both inducing diastolic arrest through membrane depolarization. STH solutions were used for the first time during cardiac surgery in 1975 and then became the most widely used crystalloid solution in open-heart surgery, until Buckberg developed his multi-dose 4:1 (4 part of crystalloid to 1 part of blood) cold blood-cardioplegia [39,40]. This hyperkalemic blood-based cardioplegia remains one of the most commonly used cardioplegic solutions worldwide [41,42]. 

## 2. Hyperkalemic Arrest: What Are the Limits?

**Figure 1:**
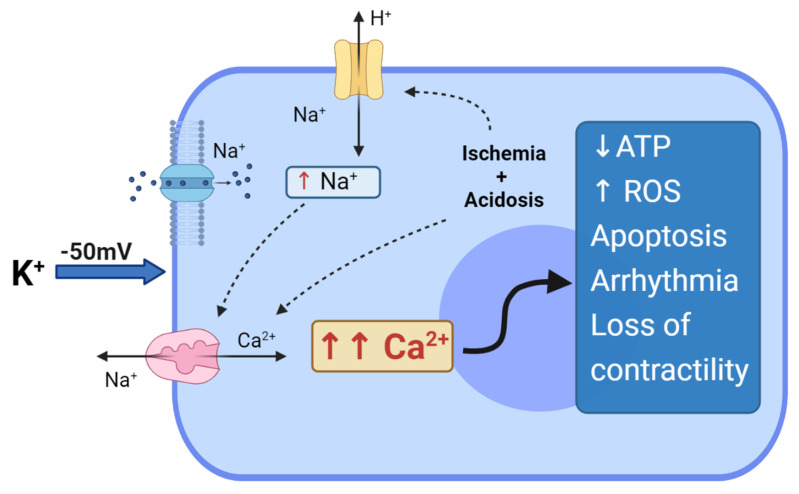
Depolarized arrest and hyperkalemia cytotoxicity.Effect of hyperkalemia and prolonged myocardial membrane depolarization at −50 mV. Na+ entry through the “window current”, which induces reversal of the Na^+^/Ca^2^^+^ exchanger with the entry of Ca^2+^ into the cell. Regional ischemia and metabolic acidosis can further impact Ca^2^^+^ loading, with increases in intracellular H^+^, thus activating the Na+/H+ exchanger; the Na+/H+ exchanger induces further activation of Na^+^/Ca^2+^ exchangers, exacerbating Ca^2+^ overloading in the myocyte. The entire cascade can induce depletion of ATP, generation of ROS, cell apoptosis, arrhythmias and loss of contractility.

The age-old problem of whether myocardial cytotoxicity and arrhythmogenicity was due to potassium ions or its anionic salt (e.g., citrate or chloride) was solved in 1975 by Tyers and colleagues [43]. Tyers’ group showed that high potassium, not the other salts, was responsible for the clinical failure of the “Melrose technique” [43]. This led to heightened scientific interest in understanding the reasons for high potassium toxicity. It was already known in the mid-1950s that increasing extracellular potassium to 16 mM leads to depolarisation of the membrane from around −80 mV to −50 mV [44]. At these potentials, the voltage-dependent Na^+^ channels are inactivated, preventing the rapid sodium-induced spike and propagation of the action potential, causing diastolic cardiac arrest. In addition, at −50 mV, the high Na^+^ driving force promotes Na^+^ entry through the Na^+^ “window currents", which remain open at these depolarized potentials. Thus, an increase in intracellular Na^+^ leads to a reversal of the voltage-dependent Na^+^/Ca^2+^ exchanger, allowing 3 Na^+^ ions extrusion in exchange for 1 Ca^2+^ ion entry, resulting in intracellular Ca^2+^ overload [45,46] (Figure 1). 

In addition to Ca^2+^ loading, myocardial ischemia and hypothermia during arrest and cross-clamping both contribute to increasing intracellular acidosis. Acidosis activates the Na^+^/H^+^ exchanger, which in turn leads to an increase in intracellular Na^+,^ inducing the activation of Na^+^/Ca^2+^ exchangers, thus resulting in further Ca^2+^ entry and cellular overload [46]. The functional deficits associated with myocyte Ca^2+^ loading at depolarised potentials is highly relevant because Ellis and colleagues showed a direct relationship between arrhythmias and high K^+^ [47] and Heyndrickx and colleagues showed a direct relationship between loss of ventricular contractility and high K^+^ [48], later to be identified as “myocardial stunning” [49,50,51]. Myocardial stunning was later confirmed by Damiano and Cohen, and Bolli and colleagues showed how it was related to Ca^2+^ overload and ATP depletion, and that rapid reperfusion led to the generation of reactive oxygen species [52,53], and the release of pro-inflammatory factors (TNF-α, NF-κb, TLRs and DAMPs) [54,55,56]. The latter are chemo-attractants for neutrophils and platelets, which are the key factors sustaining inflammation and acute vascular thrombosis [57]. Stunning is currently reported in about 10% of adults following cardiac surgery with depolarizing cardioplegic arrest; it contributes to low cardiac output syndrome, whose incidence is reported to range between 3% and 14% after CABG; furthermore, low cardiac output syndrome is associated with a from 10- to 17- fold increase in mortality [44,58].

High K^+^ is also known as a powerful vasoconstrictor [57,59], which further enhances myocardial damage, by limiting cardioplegia delivery to the myocardium [60]. This phenomenon of coronary vasocontriction has been reported frequently reported with depolarizing solutions, even after routine cardiac cases, with up to 8% of patients having coronary artery spasm (manifested by temporary ST-segment elevations on ECG after surgery), and an even greater proportion of patients exhibiting postoperative myocardial contractile dysfunction, which usually peaks 4–6 h after surgery [44].

Another important aspect of high K^+^ that is not widely appreciated is that the atria and ventricles have different sensitivities to high potassium, and promote an arrhythmogenic substrate between the chambers during reanimation after cold-diastolic arrest [44]. The atrial myocytes are the most sensitive to high K^+^, followed by ventricular cells, with the AV bundle and SA node and tracts having the lowest sensitivity, which would be further exacerbated by active warming. Different sensitivities to high K^+^ would lead to an unstable beating heart [44]. In particular, supraventricular tachyarrhythmias (mostly atrial fibrillation) have been reported in from 10% to 40–50% of patients after open cardiac surgery with depolarizing cardioplegic solutions, while life-threatening ventricular arrhythmias are considerably less common. Bradyarrhythmias caused by conduction system defects or dysfunction after cardiac procedures with depolarizing cardioplegias are frequently transient, although they sometimes require permanent pacemaker therapy (1% to 2% of patients) [61,62,63]. 

To summarize, prolonged membrane depolarization can contribute to intracellular Ca^2+^ overload and ischemia-reperfusion injury [60], which predisposes the myocardium to myocardial stunning, arrhythmias, endothelium activation, inflammation, cell apoptosis and necrosis [61,62,63,64,65,66,67,68,69]. This helps to explain the adverse effects surgeons were confronted with in the mid-1950s using the ‘Melrose technique’. Today, irrespective of efforts to use different additives, blood dilutions, and whole blood itself, the evidence suggests that depolarizing the myocardial cell membrane with from 15 to 25 mM K^+^ provides suboptimal myocardial protection, and new myocardial protective strategies are required [70].

## 3. Polarized Arrest as a Clinical Alternative

The concept of “polarized arrest” is attractive because maintaining the membrane potential close to the resting potential value (of about −80 mV) reduces the untoward effects of Ca^2+^ loading. At resting membrane potentials, Na^+^ and Ca^2+^-channels are both closed and the transmembrane fluxes are minimized; thus intracellular, Ca^2+^ overload is avoided, mitochondrial function is preserved and ATP balance maintained [70,71]. Based on these theoretical considerations, polarized arrest should confer improved myocardial preservation and recovery, avoid oxidative stress, cell death and endothelial activation during the reperfusion period. A polarised tissue is also more resistant to ischemia and inflammations reported by several in vitro and animal studies [72,73,74]. Indeed, when “inflammatory-resistance” is considered, polarizing solutions have been reported to have a greater effect on reducing porcine neutrophil priming through a greater suppression of superoxide anion generation [72]. Granfeldt and colleagues also confirmed that ALM infusion in pig models induces a reversible hypotensive and hypometabolic state, attenuates tumor necrosis factor-α levels and improves cardiac and pulmonary function [73]. Liu et al. also reported how adenosine adjunct to blood caridoplegia results in a lower troponine leakage (ischemia) as well as IL-6 secretion (inflammation) [74].

When ischemia-resistance is considered, Dobson et al. showed how the normokalemic AL polarizing concept, delivered and maintained at colder or warmer temperatures to achieve cardiac arrest and reanimation, conferred a superior post-reanimation contractile performance compared to several popular hyperkalemic formulations [44].

All of these findings refer to animal or in vitro studies, but it is reasonable to think that the membrane polarization has similar effects in human myocytes, thus providing superior myocardial protection.

### 3.1. From the Isolated Heart to Animal Studies:

**Figure 2:**
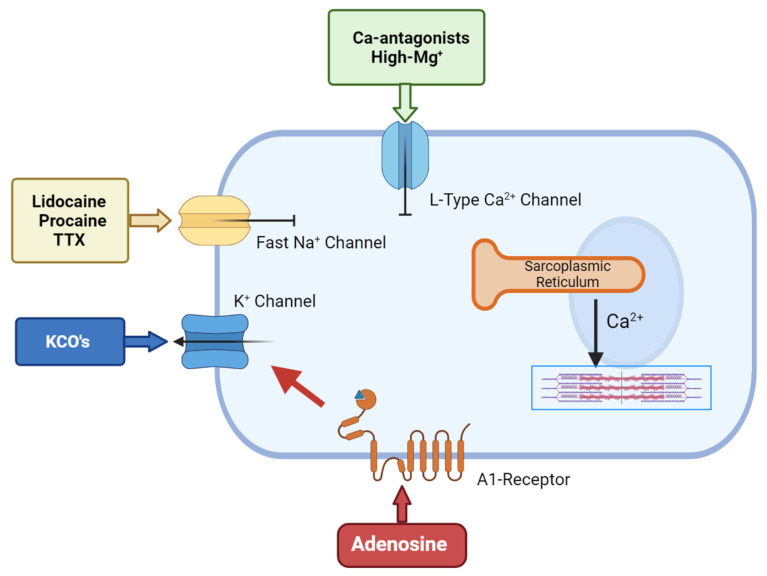
The cellular targets for hyperpolarized/polarized arrest.Adenosine-related cardioplegic effect arises from the inhibition of the sino-atrial and atrioventricular nodes, as well as from the activation of A1 receptors located on atria and ventricular cells, which open sarcolemma KATP channels to reduce action potential duration. KCO’s allows an increased potassium flux through the sarcolemma that will shift membrane potential towards the K^+^ equilibrium potential, which is normally around −90 mV in myocytes. Local anesthetic agents block the voltage-gated Na+ fast channels that are responsible for the action potential upstroke, thereby maintaining the membrane potential at/around its resting value. Ca-antagonist agents inhibit the L-type calcium channels, thus inhibiting the smooth muscle cell’s contractility.

Polarized arrest can be achieved using a number of drugs. The most common are the Na+-channel blockers such as lidocaine, procaine and tetrodotoxin (TTX) and these have been used as cardioplegic adjuncts for many years. All three block the voltage-gated Na+ fast channels that are responsible for the action potential upstroke, thereby maintaining the membrane potential at or around its resting value [75,76,77] (Figure 2). Lidocaine or procaine is often used as an additive in Bretschneider and STH solutions [32,33]. Lidocaine (and procaine) alone have not been translated as primary cardioplegic agents [36] because of their significant adverse effects (i.e., torsade de pointes and convulsions in from 1% to 8.6% during local and regional anhestesia) [76]. Similarly, although TTX has been shown to improve the post-ischemic recovery in rat hearts compared to hearts that were arrested using high K^+^ solutions, the toxicity of the drug prevents it from use in cardiac surgery [78,79,80,81,82].

Another family of polarising compounds discovered in the early 1990s are the ATP-sensitive K^+^ (K-_ATP_) channel openers [83] (Figure 2). Sarcolemma K_ATP_ channels are present in most tissues including heart and brain [76] and, upon activation, protect the cell against ischemia-reperfusion injury by lowering intracellular Ca^2+^ and preserving ATP [84,85]. At higher concentrations, K_ATP_ openers shorten the action potential duration and arrest the heart at hyperpolarized potentials [86,87]. A number of K_ATP_-channel openers have been synthesized, including nicorandil, aprikalim, pinacidil, and cromakalim [70]. Cohen and colleagues compared the cardioprotective effect of aprikalim to a hyperkalemic solution in isolated crystalloid-perfused rabbit hearts subjected to 20 min normothermic global ischemia. Hearts arrested with aprikalim recovered significantly better compared to hearts arrested by high K^+^ solution [88]. Unfortunately, like many new drugs, the application of K_ATP_ openers failed to translate from the high incidence of reperfusion arrhythmias (about 60% in animal models) [83,84,85,86,87,88].

A third alternative to high K^+^ involves non-depolarizing esmolol-HCl cardioplegia [70,89,90,91]. The idea was pioneered by Chambers and colleagues, who showed that 1 mmol/L esmolol induced a diastolic cardiac arrest in isolated rat hearts, and in the presence of oxygenated perfusate, recovered faster compared to cross-clamp fibrillation or STH hyperkalemic cardioplegic solution [92,93]. Esmolol is an ultra-short-acting cardioselective β1-blocker that inhibits the L-type calcium channels and fast Na^+^ channels, and produces a pronounced negative inotropy, slows conduction and, at high concentrations, induces polarized arrest [94,95,96,97]. However, at high concentrations, Pirk and colleagues reported prolonged infusion periods (beyond 20 min) may compromise its reversibility [95]. More recently, Chambers and colleagues reported lower concentrations of esmolol (0.6 mM) combined with adenosine (0.25 mM) alleviates this problem, with significantly improved protection compared to STH solution [96,97]. The combination of esmolol and adenosine may provide a clinically relevant polarizing cardioplegia, although further studies are required. 

Adjunctive adenosine has a long history in cardioplegia, beginning in the late 1980s. Belardinelli paved the way by demonstrating that adenosine induced hyperpolarized arrest in isolated sino-atrial (SA) node pacemaker cells [98]. This ‘cardioplegic’ effect was confirmed by Schubert et al., who compared 10 mM adenosine alone to hyperkalemic solutions in isolated rat hearts [99]. De Jong and colleagues further confirmed that high levels of adenosine induced rapid arrest; however, they also showed it delayed post-ischemic recovery, which seriously hampered its translation to humans [100]. Adenosine’s cardioplegic effect arises from inhibition of the SA and atrioventricular nodes and via activation of A1 receptors located on atria and ventricular cells, which open sarcolemma K_ATP_ channels to reduce action potential duration [44] (Figure 2). 

A question that has not been adequately addressed in the literature is why adenosine or mechanistic K_ATP_ openers have failed to translate into an effective polarising cardioplegia. In 1998, Dobson proposed that the reason for this was related to the inability of each drug to alter the upstroke of the action potential [44]. Reducing the action potential duration will arrest the heart, but, alone, it is insufficient for a coordinated return to sinus rhythm. Dobson instead proposed that combining adenosine with lidocaine may be more effective for the following: (1) shortening the action potential, and (2) inhibiting Na^+^ fast channels, which flatlines the action potential and arrests the heart at polarised potentials [44]. Magnesium was added to confer additional stability for arrest and reanimation. The Adenosine–Lidocaine–Magnesium (ALM) solution concept was borrowed from natural hibernators and developed at James Cook University, Australia [101]. The idea received proof-of-concept in isolated working rat heart preparations, followed by translation to a canine cardiopulmonary bypass model [44]. Dobson and colleagues also demonstrated the safety and superiority of ALM as a cardioplegia compared to traditional hyperkaliaemic solutions, as well as a preserving a solution even after 8 hours of cold static storage [102]. Further studies confirmed that ALM not only protects the myocardium, but also significantly reduces coronary vasculature resistance, inflammatory response, lung oedema, and prevents coagulopathy [103,104,105,106,107,108].

### 3.2. Humans Trials: Are We Getting Closer to Change?

It is remarkable that, despite 60 years of experimenting with cardioplegic solutions, there are only a handful of clinical experiences on fully-polarizing cardioplegia. Most trials have been with fully depolarising potassium solutions, with polarizing agents as additives rather than as the primary arresting agents. For example, Liu and colleagues compared standard cold blood K^+^ cardioplegia in heart valve replacement to adenosine pre-treatment plus the cardioplegia, and reported a reduced Troponin-I, IL-6 and IL-8 release and reduced myocardial injury after adenosine pre-treatment [74]. Mentzer and colleagues found a lower incidence of inotropic support in patients receiving adenosine as an additive to hyperkalemic blood cardioplegia compared with controls (the 24-h average doses for dopamine and nitroglycerine in the placebo group were 28-fold and 2.6-fold greater than their respective high-dose adenosine treatment cohorts), suggesting a better myocardial recovery in patients undergoing CABG [109]. More recently, Abdelwahab and colleagues showed that the use of adenosine immediately after aortic cross-clamping and prior to the infusion of cold hyperkalaemic cardioplegia significantly decreased postoperative Troponin-I leakage compared to standard hyperkaliemic cardioplegia alone [110]. Our Institution also conducted a trial on patients undergoing urgent CABG, and we demonstrated improved myocardial protection and functional recovery after cardioplegic arrest with the addition of polarizing ALM to a standard hyperkalemic cold blood vial [111].

Baraka and colleagues used lidocaine addition (100 mg/L) to crystalloid cardioplegic solution to prevent ventricular fibrillation after the release of the aortic cross-clamp in 50 patients undergoing CABG and in 30 patients undergoing mitral or aortic valve replacement. In the coronary artery bypass grafting group, lidocaine cardioplegia significantly reduced the incidence of reperfusion ventricular fibrillation from 100% to 42%. In the valve group, lidocaine cardioplegia also significantly reduced the incidence of reperfusion ventricular fibrillation from 93% to 42%. They reported that lidocaine cardioplegia decreased the number of direct-current countershocks required to defibrillate the heart, with no significant increase in the incidence of high-grade atrioventricular block [112]. In another trial, Ramani and colleagues compared their modified single-dose, long-acting, lidocaine-based blood cardioplegia with short-acting STH1 blood cardioplegia in patients undergoing single valve replacement. The group concluded that the method was safe and efficacious; however, there was no significant difference in creatine-phosphokinase-MB (CK-MB), Troponin-I levels, lactate level and myocardial recovery, and the study lacked statistical power to support their conclusion [113]. Finally, another randomized trial compared cardioplegia with and without lidocaine in patients undergoing CABG. Lidocaine-enriched cardioplegia significantly reduced the incidence of reperfusion ventricular fibrillation from 63% to 42%, while the incidence of atrioventricular block was higher in the lidocaine group [114].

Procaine is another local anesthetic with a long history as an additive to standard cardioplegic solutions. Mustonen et al. showed that patients undergoing CABG receiving procaine in cardioplegia had a shorter mean ventricular fibrillation time (27 ± 79 s vs. 205 ± 161 s, *p* < 0.001) and achieved stable rhythm in a higher proportion (67.6% vs. 13.5%, *p* < 0.001). Moreover, the mean number of defibrillations was lower than in patients receiving placebo (0.3 ± 0.7 vs. 2.4 ± 1.7, *p* < 0.001) [115]. Similar results were reported by Sellevold et al., who added 1mM procaine to STH2 cardioplegia in patients undergoing CABG compared to the same solution with saline adjunct. The number of synchronized direct-current shocks for conversion of atrial fibrillation was lower in the procaine group (2% vs. 100%, *p* < 0.001), as well as the post-operative myocardial enzymatic release [116].

Potassium channel openers, such as nicorandil, added to high K^+^ solutions have also been trialled in open heart surgery. Hayashi et al. compared the peri-operative results of cardioplegia with nicorandil adjunct to standard cardioplegia in patients undergoing elective CABG. The time required to achieve cardiac arrest after the initiation of cardioplegia was significantly reduced in the nicorandil group, as well as ECG-based ischemic signs during reperfusion. Furthermore, the recovery of sinus rhythm was significantly greater for nicorandil group [117]. Similar results have been showed by Chinnan et al., who added nicorandil to hyperkalaemic solution during mitral valve and CABG surgery. He reported that nicorandil did not cause significant haemodynamic changes or malignant arrhythmias in any patient [118]. Another study used nicorandil as pre-treatment before cardioplegia, confirming an enhanced myocardial protection [119].

Esmolol-based cardioplegia has been examined in a randomized single-center trial by Scorsin and colleagues on 41 patients scheduled for isolated aortic valve replacement to continuous coronary infusion with either K^+^ or esmolol during CPB [120]. Coronary glucose and lactate transmyocardial gradients were similar in both groups, indicating adequate myocardial perfusion in all patients. It was further suggested that esmolol could be effective for myocardial protection in hypertrophied hearts by reducing myocardial oxygen metabolism [120]. In another randomized single-centre trial, esmolol was used as adjunct to high K^+^ cardioplegia and it enhanced postoperative cardiac performance and reduced the postoperative troponin leakage in high-risk patients undergoing elective cardiac surgery [121,122]. In contrast, Rinne and colleagues showed that the addition of esmolol to blood cardioplegia did not increase the efficacy of cardioprotection in unstable patients during urgent coronary revascularization [123]. Therefore, the data on esmolol are conflicting and suggest further investigations in clinical practice.

In contrast, ALM cardioplegia has shown increasing promise as a fully polarizing cardioplegia in a number of human trials. The first clinical trial was conducted by Jin and colleagues involving 134 pediatric patients with low-risk congenital heart disease. The results showed cold AL crystalloid cardioplegia in pediatric patients was safe and associated with higher postoperative systolic pressures, lower blood troponin-I levels and a shortened hospitalization stays, compared with hyperkalaemic cardioplegia [124]. Jakobsen and colleagues [125] also reported that adenosine instead of high K^+^ in cold crystalloid cardioplegia (supplemented with procaine) was safe, provided a more rapid cardiac arrest, and afforded similar cardioprotection in sixty patients undergoing CABG [126]. They also reported that their adenosine and procaine solution resulted in similar hemodynamic parameters as high K^+^ but with a marked reduction in the incidence of postoperative atrial fibrillation by more than 50% [127].

To our knowledge, the only randomized human trial on full-polarizing ALM cardioplegia was conducted at our Institution in Verona Medical School. In 2016, we documented, for the first time, that “full-polarizing” ALM at high doses in a normokalemic cold blood vial was superior in humans in terms of intraoperative myocardial anaerobiosis and postoperative myocardial enzymatic leakage in both elective CABG and valve surgery, when compared to the Buckberg depolarizing cold blood solution [128]. Since then, ALM cold blood cardioplegia has been widely used at our Institution for elective adult cardiac surgery. We recently collected data from one-thousand consecutive elective adult cardiac patients (627 undergoing ALM-polarizing cardioplegia vs. 373 Buckberg cardioplegia) operated upon at our Institution over a 20-month period. These data (unpublished) confirmed significantly lower leakage of high-sensitivity troponin I (TnI_max_: ALM-POL-group: 9796 ± 8888 ng/L vs. BUCK-DEPOL-group: 12,879 ± 10,362 ng/L) during hospitalization as well as a higher spontaneous recovery of sinus rhythm at aortic declamping in ALM-group (70.9% vs. 50.9%, *p* < 0.001). These data confirm the safety of a full-polarizing cardioplegia and that the ALM solution is a strong contender for the first clinically proven alternative to high K^+^ solutions. This may be timely given that our future patients will be older, and sicker with multiple comorbidities. The future of polarized cardiac arrest is an exciting one and may play a crucial role in daily cardiac surgical practice.

## References

[1] Alexi-Meskishvili V., Böttcher W. (2011). Suturing of penetrating wounds to the heart in the nineteenth century: The beginnings of heart surgery. Ann. Thorac. Surg..

[2] Dalton H.C. (1895). Report of a Case of Stab-Wound of the Pericardium, Terminating in Recovery after Resection of a Rib and Suture of the Pericardium. Ann. Surg..

[3] Shumacker H.B. (1992). The Evolution of Cardiac Surgery.

[4] Westaby S., Bosher C. (1997). Landmarks in Cardiac Surgery.

[5] Ellis H. (2017). The early days of surgery for cardiac injuries. J. Perioper. Pract..

[6] Cohn L.H. (1993). The first successful surgical treatment of mitral stenosis: The 70th anniversary of Elliot Cutler’s mitral commissurotomy. Ann. Thorac. Surg..

[7] Cooley D.A., Frazier O.H. (2000). The past 50 years of cardiovascular surgery. Circulation.

[8] Sealy W.C. (1989). Hypothermia: Its possible role in cardiac surgery. Ann. Thorac. Surg..

[9] Lewis F.J., Taufic M. (1953). Closure of atrial septal defects with the aid of hypothermia; experimental accomplishments and the report of one successful case. Surgery.

[10] Gibbon J.H. (1978). The development of the heart-lung apparatus. Am. J. Surg..

[11] Kirklin J.W., Dushane J.W., Patrick R.T., Donald D.E., Hetzel P.S., Harshbarger H.G., Wood E.H. (1955). Intracardiac surgery with the aid of a mechanical pump-oxygenator system (gibbon type): Report of eight cases. Proc. Staff Meet. Mayo Clin..

[12] Brewer D.L., Bilbro R.H., Bartel A.G. (1973). Myocardial infarction as a complication of coronary bypass surgery. Circulation.

[13] Roberts W.C., Bulkley B.H., Morrow A.G. (1973). Pathologic anatomy of cardiac valve replacement: A study of 224 necropsy patients. Prog. Cardiovasc. Dis..

[14] Hultgren H.N., Miyagawa M., Buch W., Angell W.W. (1973). Ischemic myocardial injury during cardiopulmonary bypass surgery. Am. Heart J..

[15] Lam C.R., Gahagan T., Sergant C., Green E. (1957). Clinical experiences with Induced cardiac arrest during intracardiac surgical procedures. Ann. Surg..

[16] Ringer S. (1883). A further contribution regarding the influence of the different csonstituent of the blood on the contraction of the heart. J. Physiol..

[17] Lam C.R., Geoghegan T., Lepore A. (1955). Induced cardiac arrest for intracardiac surgical procedures; an experimental study. J. Thorac. Surg..

[18] Melrose D.G., Dreyer B., Bentall H.H., Baker J.B.E. (1955). Elective cardiac arrest. Lancet.

[19] Gerbode F., Melrose D.G. (1958). The use of K^+^ arrest in open cardiac surgery. Am. J. Surg..

[20] Effler D.B., Groves L.K., Sones F.M.J., Kolff W.J. (1956). Elective cardiac arrest in open-heart surgery; report of three cases. Cleve. Clin. Q..

[21] Schaff H.V., Dombroff R., Flaherty J.T., Bulkley B.H., Hutchins G.M., Goldman R.A., Gott V.L. (1978). Effect of K^+^ cardioplegia on myocardial ischemia and post arrest ventricular function. Circulation.

[22] Allen P., Lillehei C.W. (1957). Use of induced cardiac arrest in open heart surgery; results in seventy patients. Minn. Med..

[23] Nunn D.D., Belisle C.A., Lee W.H., Parker E.F. (1959). A comparative study of aortic occlusion alone and of K^+^ citrate arrest during cardio pulmonary bypass. Surgery.

[24] Wasserman F., Wolcott M.W., Wherrry C.G., Brodsky L. (1959). Comparative effect of 5 per cent K^+^ chloride and 30 percent K^+^ citrate in resuscitation from ventricular fibrillation following acute myocardial infarction; an experimental study. J. Thorac. Cardiovasc. Surg..

[25] Willman V.L., Cooper T., Zafiracopoulos P., Hanlon C.R. (1959). Depression of ventricular function following elective cardiac arrest with K^+^ citrate. Surgery.

[26] Bjork V.O., Fors B. (1961). Induced cardiac arrest. J. Thorac. Cardiovasc. Surg..

[27] Hufnagel C.A., Conrad P.W., Schanno J., Pifarre R. (1961). Profound cardiac hypothermia. Ann. Surg..

[28] Griepp R.B., Stinson E.B., Shumway N.E. (1973). Profound local hypothermia for myocardial protection during open-heart surgery. J. Thorac Cardiovasc. Surg..

[29] Shumway N.E., Lower R.R. (1960). Topical cardiac hypothermia for extended periods of anoxic arrest. Surg. Forum..

[30] Shumway N.E., Lower R.R., Stofer R.C. (1959). Selective hypothermia of the heart in anoxic cardiac arrest. Surg. Gynec. Obstet..

[31] Cooley D.A., Reul G.J.J., Wukasch D.C. (1975). Ischemic myocardial contracture (“stone heart”). A complication of cardiac surgery. Isr. J. Med. Sci..

[32] Bretschneider H.J. (1964). Survival time and recuperative time of the heart in normothermia and hypothermia. Verh. Dtsch. Ges. Kreislaufforsch..

[33] Bretschneider H.J., Hubner G., Knoll D., Lohr B., Nordbeck H., Spieckermann P.G. (1975). Myocardial resistance and tolerance to ischemia: Physiological and biochemical basis. J. Cardiovasc. Surg..

[34] Preusse C.J., Piper H.M., Preusse C.J. (1993). Cardioplegia with an intracellular formulation. Ischemia-Reperfusion in Cardiac Surgery.

[35] Mokbel M., Zamani H., Lei I., Chen Y.E., Romano M.A., Aaronson K.D., Haft J.W., Pagani F.D., Tang P.C. (2020). Histidine-Tryptophan-Ketoglutarate Solution for Donor Heart Preservation Is Safe for Transplantation. Ann. Thorac. Surg..

[36] Hearse D.J., Braimbridge M.V., Jynge P. (2012). Protection of the Ischemic Myocardium: Cardioplegia.

[37] Jynge P., Hearse D.J., Feuvray D., Mahalu W., Canković-Darracott S., O’Brien K., Braimbridge M.V. (1981). The St. Thomas’ hospital cardioplegic solution: A characterization in two species. Scand. J. Thorac. Cardiovasc. Surg. Suppl..

[38] Braimbridge M.V., Chayen J., Bitensky L., Hearse D.J., Jynge P., Cankovic-Darracott S. (1977). Cold cardioplegia or continuous coronary perfusion? Report on preliminary clinical experience as assessed cytochemically. J. Thorac. Cardiovasc. Surg..

[39] Buckberg G.D. (1979). A proposed “solution” to the cardioplegic controversy. J. Thorac. Cardiovasc. Surg..

[40] Buckberg G.D. (1989). Antegrade/retrograde blood cardioplegia to ensure cardioplegic distribution: Operative techniques and objectives. J. Card. Surg..

[41] Karthik S., Grayson A.D., Oo A.Y., Fabri B.M. (2004). A survey of current myocardial protection practices during coronary artery bypass grafting. Ann. R Coll. Surg. Engl..

[42] Ali J.M., Miles L.F., Abu-Omar Y., Galhardo C., Falter F. (2018). Global Cardioplegia Practices: Results from the Global Cardiopulmonary Bypass Survey. J. Extra. Corpor. Technol..

[43] Tyers G.F., Todd G.J., Niebauer I.M., Manley N.J., Waldhausen J.A. (1975). The mechanism of myocardial damage following K^+^ citrate (Melrose) cardioplegia. Surgery..

[44] Dobson G.P., Faggian G., Onorati F., Vinten-Johansen J. (2013). Hyperkalemic cardioplegia for adult and pediatric surgery: End of an era?. Front. Physiol..

[45] Bers D.M., Despa S. (2006). Cardiac myocytes Ca^2+^ and Na+ regulation in normal and failing hearts. J. Pharmacol. Sci..

[46] Bers D.M., Despa S. (2009). Na+ transport in cardiac myocytes; Implications for excitation-contraction coupling. IUBMB Life.

[47] Ellis R.J., Mavroudis C., Gardner C., Turley K., Ullyot D., Ebert P.A. (1980). Relationship between atrioventricular arrhythmias and the concentration of K^+^ ion in cardioplegic solution. J. Thorac. Cardiovasc. Surg..

[48] Heyndrickx G.R., Millard R.W., McRitchie R.J., Maroko P.R., Vatner S.F. (1975). Regional myocardial functional and electrophysiological alterations after brief coronary artery occlusion in conscious dogs. J. Clin. Invest..

[49] Tsutsumi T., Wyatt R.F., Abildskov J.A. (1983). Effects of hyperkalemia on local changes of repolarization duration in canine left ventricle. J. Electrocardiol..

[50] Braunwald E., Kloner R.A. (1982). The stunned myocardium: Prolonged, postischemic ventricular dysfunction. Circulation.

[51] Kloner R.A., Przyklenk K., Kay G.L. (1994). Clinical evidence for stunned myocardium after coronary artery bypass surgery. J. Card Surg..

[52] Damiano R.J., Cohen N.M. (1994). Hyperpolarized arrest attenuates myocardial stunning following global surgical ischemia: An alternative to traditional hyperkalemic cardioplegia?. J. Card Surg..

[53] Bolli R., Patel B.S., Jeroudi M.O., Lai E.K., McCay P.B. (1988). Demonstration of free radical generation in "stunned" myocardium of intact dogs with the use of the spin trap alpha-phenyl N-tert-butyl nitrone. J. Clin. Invest..

[54] Parolari A., Rubini P., Cannata A., Bonati L., Alamanni F., Tremoli E., Biglioli P. (2002). Endothelial damage during myocardial preservation and storage. Ann. Thorac. Surg..

[55] Verma S., Anderson T.J. (2002). Fundamentals of endothelial function for the clinical cardiologist. Circulation..

[56] Higashi Y., Noma K., Yoshizumi M., Kihara Y. (2009). Endothelial function and oxidative stress in cardiovascular diseases. Circ. J..

[57] Siess W. (1989). Molecular mechanisms of platelet activation. Physiol. Rev..

[58] Algarni K.D., Maganti M., Yau T.M. (2011). Predictors of low cardiac output syndrome after isolated coronary artery bypass surgery:trends over 20 years. Ann. Thorac. Surg..

[59] Sellke F.W., Boyle E.M., Verrier E.D. (1996). Endothelial cell injury in cardiovascular surgery: The pathophysiology of vasomotor dysfunction. Ann. Thorac. Surg..

[60] He G.W. (2005). Endothelial function related to vascular tone in cardiac surgery. Heart Lung Circ..

[61] Conti V.R., Ware D.L. (2002). Cardiac arrhythmias in cardiothoracic surgery. Chest Surg. Clin. N Am..

[62] Gundry S.R., Sequeira A., Coughlin T.R., McLaughlin J.S. (1989). Postoperative conduction disturbances: A comparison of blood and crystalloid cardioplegia. Ann. Thorac. Surg..

[63] Rousou J.A., Meeran M.K., Engelman R.M., Breyer R.H., Lemeshow S. (1985). Does the type of venous drainage or cardioplegia affect postoperative conduction and atrial arrhythmias?. Circulation.

[64] Chambers D.J., Hearse D.J., Sperelakis N., Kurachi Y., Terzic A., Cohen M.V. (2001). Cardioplegia and surgical ischemia. Heart Physiology and Pathophysiology.

[65] Lubbe W.F., Podzuweit T., Opie L.H. (1992). Potential arrhythmogenic role of cyclic adenosine monophosphate (AMP) and cytosolic calcium overload: Implications for prophylactic effects of beta-blockers in myocardial infarction and proarrhythmic effects of phosphodiesterase inhibitors. J. Am. Coll. Cardiol..

[66] Paterson D.J., Rogers J., Powell T., Brown H.F. (1993). Effect of catecholamines on the ventricular myocyte action potential in raised extracellular K^+^. Acta. Physiol. Scand..

[67] Vittone L., Said M., Mattiazzi A. (2006). beta 2-Adrenergic stimulation is involved in the contractile dysfunction of the stunned heart. Naunyn. Schmiedebergs. Arch. Pharmacol..

[68] Lameris T.W., de Zeeuw S., Alberts G., Boomsma F., Duncker D.J., Verdouw P.D., Veld A.J., van Den Meiracker A.H. (2000). Time course and mechanism of myocardial catecholamine release during transient ischemia in vivo. Circulation.

[69] Yang Q., He G.W. (2005). Effect of cardioplegic and organ preservation solutions and their components on coronary endothelium-derived relaxing factors. Ann. Thorac. Surg..

[70] Chambers D.J. (2003). Mechanisms and alternative methods of achieving cardiac arrest. Ann. Thorac. Surg..

[71] Dobson G.P. (2010). Membrane polarity: A target for myocardial protection and reduced inflammation in adult and pediatric cardiothoracic surgery. J. Thorac. Cardiovasc. Surg..

[72] Shi W., Jiang R., Dobson G.P., Vinten-Johansen J. (2012). The novel non-depolarizing, normokalemic cardioplegia formulation adenosine–lidocaine (Adenocaine) exerts superior anti-neutrophil effects by synergistic actions of its components. J. Thorac. Cardiovasc. Surg..

[73] Granfeldt A., Letson H.L., Dobson G.P., Shi W., Vinten-Johansen J., Tønnesen E. (2014). Adenosine, lidocaine and Mg2+ improves cardiac and pulmonary function, induces reversible hypotension and exerts anti-inflammatory effects in an endotoxemic porcine model. Crit. Care..

[74] Liu R., Xing J., Miao N., Li W., Liu W., Lai Y.-Q., Luo Y., Ji B. (2009). The myocardial protective effect of adenosine as an adjunct to intermittent blood cardioplegia during open heart surgery. Eur. J. Cardio-Thorac. Surg..

[75] Snabaitis A.K., Shattock M.J., Chambers D.J. (1997). Comparison of polarized and depolarized arrest in the isolated rat heart for long-term preservation. Circulation.

[76] Sternbergh W.C., Brunsting L.A., Abd-Elfattah A.S., Wechsler A.S. (1989). Basal metabolic energy requirements of polarized and depolarized arrest in rat heart. Am. J. Physiol..

[77] Miller R.D., Katzung B.G. (1998). Local anesthetics. Basic and Clinical Pharmacology.

[78] Brown D.L., Ransom D.M., Hall J.A., Leicht C.H., Schroeder D.R., Offord K.P. (1995). Regional anesthesia and local anesthetic-induced systemic toxicity: Seizure frequency and accompanying cardiovascular changes. Anesth. Analg..

[79] Attwell D., Cohen I., Eisner D., Ohba M., Ojeda C. (1979). The steady state TTX-sensitive (‘window’) Na+ current in cardiac Purkinje fibres. Pflugers Arch..

[80] Tyers G.F., Todd G.J., Niebauer I.M., Manley N.J., Waldhausen J.A. (1974). Effect of intracoronary tetrodotoxin on recovery of the isolated working rat heart from sixty minutes of ischemia. Circulation.

[81] Tyers G.F., Todd G.J., Neely J.R., Waldhausen J.A. (1975). The mechanism of myocardial protection from ischemic arrest by intracoronary tetrodotoxin administration. J. Thorac. Cardiovasc. Surg..

[82] Narahashi T. (2008). Tetrodotoxin: A brief history. Proc. Jpn. Acad. Ser. B Phys. Biol. Sci..

[83] Noma A. (1983). ATP-regulated K^+^ channels in cardiac muscle. Nature.

[84] Hearse D.J. (1995). Activation of ATP-sensitive K^+^ channels: A novel pharmacological approach to myocardial protection?. Cardiovasc. Res..

[85] Nichols C.G. (2016). Adenosine Triphosphate-Sensitive Potassium Currents in Heart Disease and Cardioprotection. Card Electrophysiol. Clin..

[86] McPherson C.D., Pierce G.N., Cole W.C. (1993). Ischemic cardioprotection by ATP-sensitive K1 channels involves high-energy phosphate preservation. Am. J. Physiol..

[87] Cohen N.M., Wise R.M., Wechsler A.S., Damiano R.J. (1993). Elective cardiac arrest with a hyperpolarizing adenosine triphosphate-sensitive K^+^ channel opener. A novel form of myocardial protection?. J. Thorac. Cardiovasc. Surg..

[88] Chi L., Uprichard A.C., Lucchesi B.R. (1990). Profibrillatory actions of pinacidil in a conscious canine model of sudden coronary death. J. Cardiovasc. Pharmacol..

[89] Bessho R., Chambers D.J. (2001). Myocardial protection: The efficacy of an ultrashort-acting beta-blocker, esmolol, as a cardioplegic agent. J. Thorac. Cardiovasc. Surg..

[90] Bessho R., Chambers D.J. (2002). Myocardial protection with oxygenated esmolol cardioplegia during prolonged normothermic ischemia in the rat. J. Thorac. Cardiovasc. Surg..

[91] Arlock P., Wohlfart B., Sjoberg T., Steen S. (2005). The negative inotropic effect of esmolol on isolated cardiac muscle. Scand. Cardiovasc. J..

[92] Fallouh H.B., McLatchie L.M., Shattock M.J., Chambers D.J., Kentish J.C. (2007). Esmolol as a cardioplegic agent: An effect beyond (beta)-blockade. Circulation.

[93] Fallouh H.B., McLatchie L.M., Bardswell S.C., Shattock M.J., Chambers D.J., Kentish J.C. (2008). Myocardial arrest by esmolol: Negative inotropy induced by calcium and sodium channel blockade. J. Mol. Cell. Cardiol..

[94] Deng C.Y., Lin S.G., Zhang W.C., Kuang S.J., Qian W.M., Wu S.L., Shan Z.X., Yang M., Yu X.Y. (2006). Esmolol inhibits Na+ current in rat ventricular myocytes. Methods Find. Exp. Clin. Pharmacol..

[95] Pirk J., Kolár F., Ost’ádal B., Sedivý J., Stambergová A., Kellovský P. (1999). The effect of the ultrashort beta-blocker esmolol on cardiac function recovery: An experimental study. Eur. J. Cardio-Thorac. Surg..

[96] Fujii M., Chambers D.J. (2013). Cardioprotection with esmolol cardioplegia: Efficacy as a blood-based solution. Eur. J. Cardio-Thorac. Surg..

[97] Nishina D., Chambers D.J. (2018). Efficacy of esmolol cardioplegia during hypothermic ischaemia. Eur. J. Cardio-Thorac. Surg..

[98] Belardinelli L., Giles W.R., West A. (1988). Ionic mechanisms o adenosine actions in pacemaker cells from rabbit heart. J. Physiol..

[99] Schubert T., Vetter H., Owen P., Reichart B., Opie L.H. (1989). Adenosine cardioplegia. Adenosine versus K^+^ cardioplegia:effects on cardiac arrest and postischemic recovery in the isolated rat heart. J. Thorac. Cardiovasc. Surg..

[100] De Jong J.W., van der Meer P., van Loon H., Owen P., Opie L.H. (1990). Adenosine as adjunct to K^+^ cardioplegia: Effect on function, energy metabolism, and electrophysiology. J. Thorac. Cardiovasc. Surg..

[101] Dobson G.P. (2004). Organ arrest, protection and preservation: Natural hibernation to cardiac surgery. Comp. Biochem. Physiol. B Biochem. Mol. Biol..

[102] Rudd D.M., Dobson G.P. (2011). Eight hours of cold static storage with adenosine and lidocaine (Adenocaine) heart preservation solutions: Toward therapeutic suspended animation. J. Thorac. Cardiovasc. Surg..

[103] Griffin M.J., Letson H.L., Dobson G.P. (2014). Adenosine, lidocaine and Mg2+ (ALM) induces a reversible hypotensive state, reduces lung edema and prevents coagulopathy in the rat model of polymicrobial sepsis. J. Trauma Acute Care Surg..

[104] Vinten-Johansen J., Thourani V.H., Ronson R.S., Jordan J.E., Zhao Z.Q., Nakamura M., Velez D., Guyton R.A. (1999). Broad-spectrum cardioprotection with adenosine. Ann. Thorac. Surg..

[105] Boehm D.H., Human P.A., von Oppell U., Owen P., Reichenspurner H., Opie L.H., Rose A.G., Reichart B. (1991). Adenosine cardioplegia: Reducing reperfusion injury of the ischaemic myocardium?. Eur. J. Cardio-Thorac. Surg..

[106] Dobson G.P., Jones M.W. (2004). Adenosine and lidocaine: A new concept in non depolarizing surgical myocardial arrest, protection, and preservation. J. Thorac. Cardiovasc. Surg..

[107] Djabir Y., Letson H.L., Dobson G.P. (2013). Adenosine, lidocaine, and Mg2+ (ALM™) increases survival and corrects coagulopathy after eight-minute asphyxial cardiac arrest in the rat. Shock.

[108] Sloots K.L., Dobson G.P. (2010). Normokalemic adenosine-lidocaine cardioplegia: Importance of maintaining a polarized myocardium for optimal arrest and reanimation. J. Thorac. Cardiovasc. Surg..

[109] Mentzer R.M., Rahko P.S., Molina-Viamonte V., Canver C.C., Chopra P.S., Love R.B., Cook T.D., Hegge J.O., Lasley R.D. (1997). Safety, tolerance, and efficacy of adenosine as an additive to blood cardioplegia in humans during coronary artery bypass surgery. Am. J. Cardiol..

[110] Abdelwahab A.A., Sabry M., Elshora H.A., Arafat A.A. (2019). Effect of Fast Cardioplegic Arrest Induced by Adenosine on Cardiac Troponin Levels After Heart Valve Surgery. Heart Lung Circ..

[111] Onorati F., Santini F., Dandale R., Ucci G., Pechlivanidis K., Menon T., Chiominto B., Mazzucco A., Faggian G. (2013). “Polarizing” microplegia improves cardiac cycle efficiency after CABG for unstable angina. Int. J. Cardiol..

[112] Baraka A., Hirt N., Dabbous A., Taha S., Rouhana C., el-Khoury N., Ghabash M., Jamhoury M., Sibaii A. (1993). Lidocaine cardioplegia for prevention of reperfusion ventricular fibrillation. Ann. Thorac. Surg..

[113] Ramani J., Malhotra A., Wadhwa V., Sharma P., Garg P., Tarsaria M., Pandya H. (2017). Single-Dose Lignocaine-Based Blood Cardioplegia in Single Valve Replacement Patients. Braz. J. Cardiovasc. Surg..

[114] Wallace S.R., Baker A.B. (1994). Incidence of ventricular fibrillation after aortic cross-clamp release using lignocaine cardioplegia. Anaesth. Intensive Care.

[115] Mustonen P.K., Hippeläinen M.J., Pöyhönen M.J., Rehnberg L.S. (1996). Procaine and timing of aortic declamping affect ventricular reperfusion fibrillation. Ann. Chir. Gynaecol..

[116] Sellevold O.F., Berg E.M., Levang O.W. (1995). Procaine is effective for minimizing postischemic ventricular fibrillation in cardiac surgery. Anesth. Analg..

[117] Hayashi Y., Sawa Y., Ohtake S., Nishimura M., Ichikawa H., Matsuda H. (2001). Controlled nicorandil administration for myocardial protection during coronary artery bypass grafting under cardiopulmonary bypass. J. Cardiovasc. Pharmacol..

[118] Chinnan N.K., Puri G.D., Thingnam S.K. (2007). Myocardial protection by nicorandil during open-heart surgery under cardiopulmonary bypass. Eur. J. Anaesthesiol..

[119] Li Y., Iguchi A., Tsuru Y., Nakame T., Satou K., Tabayashi K. (2000). Nicorandil pretreatment and improved myocardial protection during cold blood cardioplegia. Jpn. J. Thorac. Cardiovasc. Surg..

[120] Scorsin M., Mebazaa A., Al Attar N., Medini B., Callebert J., Raffoul R., Ramadan R., Maillet J.M., Ruffenach A., Simoneau F. (2003). Efficacy of esmolol as a myocardial protective agent during continuous retrograde blood cardioplegia. J. Thorac. Cardiovasc. Surg..

[121] Zangrillo A., Bignami E., Noè B., Nardelli P., Licheri M., Gerli C., Crivellari M., Oriani A., Di Prima A.L., Fominskiy E. (2021). Esmolol in Cardiac Surgery: A Randomized Controlled Trial. J. Cardio-Thorac. Vasc. Anesth..

[122] Bignami E., Guarnieri M., Franco A., Gerli C., De Luca M., Monaco F., Landoni G., Zangrillo A. (2017). Esmolol before cardioplegia and as cardioplegia adjuvant reduces cardiac troponin release after cardiac surgery. A Randomized Trial. Perfusion..

[123] Rinne T., Harmoinen A., Kaukinen S. (2000). Esmolol cardioplegia in unstable coronary revascularisation patients. A randomised clinical trial. Acta Anaesthesiol. Scand..

[124] Jin Z.X., Zhang S.L., Wang X.M., Bi S.H., Xin M., Zhou J.J., Cui Q., Duan W.X., Wang H.B., Yi D.H. (2008). The myocardial protective effects of a moderate-K^+^ adenosine-lidocaine cardioplegia in pediatric cardiac surgery. J. Thorac. Cardiovasc. Surg..

[125] Jakobsen Ø., Næsheim T., Aas K.N., Sørlie D., Steensrud T. (2013). Adenosine instead of supranormal K^+^ in cardioplegia: It is safe, efficient, and reduces the incidence of postoperative atrial fibrillation. A randomized clinical trial. J. Thorac. Cardiovasc. Surg..

[126] Jakobsen Ø., Muller S., Aarsaether E., Steensrud T., Sørlie D.G. (2007). Adenosine instead of supranormal K^+^ in cardioplegic solution improves cardioprotection. Eur. J. Cardio-Thorac. Surg..

[127] Vinten-Johansen J., Dobson G.P. (2013). Adenosine-procaine cardioplegia and adenosine-lidocaine cardioplegia: Two sides of the same coin?. J. Thorac. Cardiovasc. Surg..

[128] Onorati F., Dobson G.P., San Biagio L., Abbasciano R., Fanti D., Covajes C., Menon T., Gottin L., Biancari F., Mazzucco A. (2016). Superior Myocardial Protection Using "Polarizing" Adenosine, Lidocaine, and Mg2+ Cardioplegia in Humans. J. Am. Coll. Cardiol..

